# Impact of COVID-19 on depression and anxiety among healthcare professionals in Abu Dhabi

**DOI:** 10.1371/journal.pone.0282198

**Published:** 2023-03-31

**Authors:** Amal Abdul Rahim Al Zarooni, Aljazia Khalfan Alghfeli, Hamda Musabbah Alremeithi, Roqayah Abdulla Almadhaani, Latifa Baynouna Alketbi

**Affiliations:** Ambulatory Healthcare Services, Abu Dhabi Health Services Company, Abu Dhabi, United Arab Emirates; University of Science and Technology of Fujairah, YEMEN

## Abstract

**Background:**

COVID-19 has affected Healthcare workers in many ways. One of the important areas is the psychological impact. This study aims to examine the effects of the COVID-19 outbreak on the mental health of Healthcare Professionals (HCP) and associated factors.

**Methods:**

A cross-sectional study was conducted among healthcare providers in the Emirates of Abu Dhabi, United Arab Emirates, between April 11^th^, and July 23^rd^, 2020. The study was conducted by using an online anonymous Self-administered questionnaire through the survey monkey platform. A convenient sampling method was used to distribute the online survey link through the organization’s email network admin list and smartphone messaging. Descriptive statistics, t-tests, and multivariant linear regression were used.

**Results:**

Anxiety and depression risk scores were measured in a total of 1268 participants of healthcare providers. More than half of the participants reported symptoms of anxiety (51.5%). Depression symptoms were revealed in 38.3% of participating providers. A mild risk of anxiety was reported in 28.8% of the participants and 12.68% of the participants registered moderate anxiety risk scores and 9.95% reported a severe risk of anxiety. Among all participants, 4.3% and 2.7% reported moderately severe and severe risk of depression, respectively, while 22.5% and 8.8% of the participating healthcare providers documented mild and moderate depression risk. Anxiety and depression risk scores were significantly and negatively associated with age and working in primary health care.

**Conclusions:**

The high prevalence of anxiety and depression recorded among HCP during the pandemic suggests that mental health intervention and support are necessary to ensure the psychological well-being of HCP.

## Introduction

The Ongoing infections of COVID-19 have created a global health burden and public health problems in many countries [1]. COVID-19 was announced as a global pandemic on March 11, 2020, by the World Health Organization (WHO) [2]. COVID-19 is a highly contagious virus that causes mild to severe respiratory tract infections [3]. More than 700 000 COVID-19 cases had been reported in the United Arab Emirates (UAE) by August 2021. Nevertheless, the UAE had one of the lowest reported death rates (0.29%) in the world [4,5].

In the United Arab Emirates (UAE), changes in the healthcare system have been adapted to the public health needs caused by the COVID-19 pandemic. Currently, healthcare providers are making remarkable efforts to control additional outbreaks of the disease [1]. Healthcare providers are on the front lines during COVID-19 with a higher risk of exposure to and acquiring COVID-19 infection, this may expose them to mental distress causing anxiety, depression, and emotional stress [6]. Providing direct care to patients with COVID-19, or being required to undergo quarantine or isolation, may lead to psychological distress in healthcare providers [7]. In UAE the COVID-19 vaccine was available to frontline healthcare providers in September 2020 [4]. Qualitative data from frontline staff in the UAE showed that they were experiencing COVID-19-related stress, as a result of their working in a hazardous environment and due to the trauma of the amount of death seen initially [8–10]. A national study has found that levels of anxiety and depression are significantly higher than those reported in previous pre-pandemic studies [11]. A study found that COVID-19 has a significant psychological impact on adults and children in the UAE [12]. HCWs in the UAE reported a high prevalence of psychological distress and anxiety when responding to the challenges of COVID-19 [13,14]. Infectious disease outbreaks are likely to affect the psychological health of healthcare workers (HCWs) practicing on the front lines of the pandemic [15]. Initial evidence that a considerable proportion of HCWs experienced mood and sleep disturbances during the outbreak, justifies the recommendation to establish methods to mitigate mental health risks and adjust interventions under the conditions of the pandemic [16]. Healthcare workers contributed significantly to controlling the infectious disease, in this context psychologist support, mental counseling, and helpline support were provided to the healthcare team in the United Arab Emirates. The psychological impact of COVID-19 on (UAE) healthcare providers is not studied extensively. The current study aimed to examine the effects of the COVID-19 outbreak on the manifestation of depression and anxiety in HCWs and associated factors.

## Methods

### Study design

A cross-sectional study. Data were collected using an anonymous online self-administered questionnaire, written in English. A convenient sampling method was used to distribute the online survey link through the organization’s email network admin list and smartphone messaging. The survey monkey platform was used.

### Setting

A study was conducted in Abu Dhabi, United Arab Emirates, among healthcare providers from primary healthcare centers and inpatient hospitals in the private and public sectors. The study was conducted between 11 April to 23 July.

### Participants

Participants were eligible if they were 18 years of age or older, health care providers, and had access to the Internet via computer and/or smartphone. Participants who did not sign the e-consent were excluded.

### Variables

The survey was designed to collect information regarding the demographic data of the participants, including age, sex, occupation, specialty, years of experience, the city, and the setting of their practice. Screening for Depression and anxiety using PHQ-9 And GAD-7 Questionnaire were done. The questionnaire was piloted among 20 healthcare providers.

### Measurements

Depression screening was accomplished using the Nine-Item Patient Health Questionnaire (PHQ-9) [17]. PHQ-9 is an effective and reliable tool for screening as well as for monitoring the severity of depression [5]. It has been widely used in community-based settings, in the general population, and in primary care [18,19]. In a meta-analysis, the reliability and validity of PHQ-9 were found to be better than that of DSM-IV (Diagnostic and Statistical Manual of Mental Disorders, fourth edition) [18,20]. The PHQ-9 includes nine items, each requiring four responses. Each response is scored according to the following: not at all = 0, several days = 1, more than half a day = 2, almost every day = 3. The total depression score may range from 0–27. A total score of 0–4 indicates a risk of minimal depression, with a score of 5–9 signifying a mild risk of depression. A total score of 10–14 indicates a moderate risk of depression, with scores of 15–19, and 20–27, demonstrating the moderately severe and severe risk of depression, respectively.

The Generalized Anxiety Disorder scoring system (GAD7) includes a 7-item questionnaire [21]. Evidence supports the reliability and validity of the GAD-7 as a measure of anxiety in the general population [22]. GAD-7 has also shown strong psychometric properties in different populations [23]. Each item requires four responses, and each response is scored as follows: not at all = 0, several days = 1, more than half a day = 2, almost every day = 3. The total Anxiety score may range from 0–21. A total score of 0–4 indicates a minimal risk of anxiety, with a total score of 5–9 demonstrating a mild risk of anxiety. Total scores of 10–14, and 15–21 indicate moderate and severe risk of anxiety, respectively.

### Study size

The sample size was determined using a margin of error of 5%, and a confidence interval (CI) of 95%. The minimum required sample size was 383. Due to the distribution of the survey as online sampling, it was a convenient sampling method.

### Statistical analysis

Data were analyzed using the SPSS version 21 Software program. Descriptive statistics such as mean, and standard deviation (SD) were computed for quantitative variables and frequencies, and percentages were calculated for categorical variables. Multivariate Linear regression analyses were conducted to determine the determinants. A significant level of p-value ≤0.05 was used.

### Ethics and confidentiality

All study participants were completely informed about the purpose, methods, time frame, and role of the study. Online consent from the participants for using their data for research was established before enrolment. The study was approved by the Ambulatory Health care services human ethics committee and the Abu Dhabi health care service central human Ethics Committee Reference: DOH/CVDC/2021/172.

The study was conducted following the Ethics Committee’s guidelines. The questionnaire was anonymous to the participants and did not record any participants’ identifiers or personal information. The confidentiality of the study participants was maintained.

## Results

The survey was opened by 2371 (1268, 53.5%) healthcare providers who consented and completed the online survey, (738,58.2%) of whom were working in in-patient hospitals, (306,24.1%) in primary healthcare practices, and (210, 16.6%) were working in emergency and ICU care settings. The data was collected during the COVID-19 pandemic, between April 11^th^ and July 23^rd^, 2020. Most of the participants (1124, 88.6%) were from SEHA, and only (144, 11.4%) originated from outside SEHA. The mean age of the participants was 40.26 (SD 8.85) and was mostly female (945,74.5%). Nurses constituted (785, 61.9%) of the respondents, and (234,18.4%) were Physicians including consulting physicians, specialists, and resident doctors. For allied health professionals, technicians comprised (187,14.7%) and only (62, 4.9%) were pharmacists. Physicians who enrolled represented various specialties, including family medicine (79, 6.2%), internal medicine (100, 7.9%), Obstetrics and gynecology (105,8.3%), Pediatrics (141, 1.1%), Psychiatry (21,1.7%), and Surgeons (120, 9.5%). Most participants originated from Abu Dhabi and Al Ain (793, 62.5%), (331, 26.1%) respectively, and 9.9% of participants hailed from the western region. The mean work experience was 14.3 years (SD 8.28) (Table 1).

**Table 1:** Characteristics of the study population.

Age years (SD)	Mean age	40 .26 years (8.85)
Sex % (N)	Female Male	74.5 (945) 25.5 (323)
Occupation % (N)	Physicians Nurse Allied health professional	18.5 (234) 61.9 (785) 19.6 (249)
Specialty % (N)	Family medicine Internal Medicine Obstetrics and Gynecology Other Pediatrics Psychiatry Surgical specialties	6.2 (79) 7.9 (100) 8.3 (105) 55.4 (702) 11.1 (141) 1.7 (21) 9.5 (120)
Practice sitting %	Emergency /ICU In-patient hospital-based Other Primary health care clinic	16.6 (210) 58.2 (738) 1.1 (14) 24.1 (306)
City %	Abu Dhabi Alain Other Western Region	62.5 (793) 26.1 (331) 1.4 (18) 9.9 (125)
Experience (SD)	Mean of years of work experience	14.3 years (8.28)

Anxiety risk scores were measured for each of the 1,268 participants of healthcare providers. Generalized anxiety scores were measured using Generalized Anxiety Disorder-7. More than half of the participants (51.5%) reported anxiety symptoms, and almost half (48.48%) scored in the minimal anxiety risk category. A mild risk of anxiety was reported in 28.8% of all healthcare providers, and 12.68% of respondents revealed moderate anxiety risk scores, with 9.95% reporting severe anxiety risk.

Mild and moderate anxiety risks (22.6%, 9.9%) were noted among female healthcare providers. A slightly higher severe anxiety risk (6.8%) was noted among male healthcare providers although this was not significant.

It was noted that anxiety risk scores decreased with additional years of experience. Only 3.6% of healthcare providers with more than 30 years of experience reported severe anxiety risk while 16% of HCP with less than 5 years of experience had scores indicating severe anxiety risk.

Mild anxiety risk was primarily observed among healthcare providers between 41–50 years of age (31.3%), while moderate anxiety risk was noted among healthcare providers who were beyond 50 years of age (16.2%).

Anxiety risk scores were significantly associated with occupation, p = 0.02. Mild anxiety risk affected more than 30% of the Nursing (30.2%) and physicians (30.2%), and resident doctors (36.1%) occupations. Moderate anxiety risk was mostly noted among consultants (15.6%) and nursing (14.8%) and severe anxiety risk were significantly noted among resident doctors (19.4%).

Anxiety risk scores were significantly associated with specialties, and the p-value was 0.029. Mild anxiety risk affected more than 30% of the Psychiatry (30%) Family medicine (30.4%), Pediatrics (31.9%), Obstetrics and gynecology, (35.6%), and Internal medicine (36.4%) specialties. Moderate anxiety risk was mostly noted in Obstetrics and gynecology (18.3%). Severe anxiety risk scores were the highest among Internal medicine (16.2%) specialists (Table 2).

**Table 2:** Anxiety risk scores and prevalence among healthcare providers in correlation with HCP gender, years of experience, age group, occupation, and specialty.

Variable	Mild Anxiety Risk % (N)	Moderate Anxiety Risk % (N)	Severe Anxiety Risk % (N)	P value
**Gender**				0.064
Female HCP	30.4 (281)	13.3 (123)	9.2 (85)	
Male HCP	24.6 (79)	10.9 (35)	12.1 (39)	
**Years of experience**				0.074
less than 5	20.6 (36)	12 (21)	16 (28)	
6–10	32.4 (101)	10.9 (34)	9.3 (29)	
11–20	31(157)	13.2 (67)	9.5 (48)	
21–30	25.5 (50)	14.8 (29)	8.7 (17)	
more than 30	28.6 (16)	12.5 (7)	3.6(2)	
**Age group**				0.073
less than 30	28.3 (45)	13.8 (22)	14.5 (23)	
30–40	29.8 (157)	11.8 (62)	11 (58)	
41–50	31.3 (110)	11.4 (40)	8 (28)	
more than 50	22.9 (48)	16.2 (34)	7.1 (15)	
**Occupation**				0.02
Physicians	30.2 (70)	12.5 (29)	9.1 (21)	
Nursing	30.2 (233)	14.8 (114)	9.2 (17)	
Allied health professional	23.5 (57)	6.2 (15)	13.2(32)	
**Specialty**				0.029
Family medicine	30.4 (24)	11.4 (9)	2.5 (2)	
Internal medicine	36.4 (36)	12.1 (120	16.2 (160	
Obstetrics and Gynecology	35.6 (37)	18.3 (19)	6.7 (7)	
Pediatric	31.9 (44)	16.7 (23)	8.7 (12)	
Psychiatry	30 (6)	5 (1)	10 (2)	
Surgery	25 (30)	10.8 (13)	9.2 (11)	
Others	26.7 (183)	11.8 (81)	10.8 (74)	
**Practice sitting**				0.203
Emergency and ICU care	26.8 (56)	14.8 (31)	12.4 (26)	
In-patient hospital	28.8(209)	12.8 (93)	10.8 (26)	
Other	33.3 (4)	16.7(2)	16.7 (2)	
Primary health care	30.3(91)	10.7 (32)	6 (18)	

*Generalized anxiety disorder scoring system included 7 items. Each item has four responses. Each response had a certain score as the following: Not at all = 0, several days = 1, more than half a day = 2, almost every day = 3 anxiety items total score ranged from 0–21. Total score of 0–4 indicates minimal anxiety. A total score of 5–9 indicates mild anxiety. A total score of 10–14 indicates moderate anxiety. A total score of 15–21 indicates severe anxiety.

Depression risk scores were measured for each of the 1,268 participating healthcare providers. Depression symptoms were reported in 38.3% of respondents. Most of the participants who reported a score above 4 were with minimal depression risk scores in the category of mild risk, 5–9, (60.3%). Moderately severe depression risk was reported in (4.3%) of the total participants and severe depression risk was reported in (2.7%) of them. HCPs reported 22.5% and 8.8% in mild and moderate risk of depression, respectively.

Mild and moderate depression risk (23.5% and 9.1%) respectively, were observed more frequently among females. Moderately severe (6.6%) and severe depression risk rates (3.5%) were higher among male healthcare providers. There was no significant association between the gender of the healthcare provider and the severity of depression risk (p = 0.119).

Mild, moderate, moderately severe, and severe depression risks were mostly noted among healthcare providers aged less than 30 (23.9%, 13.2%, 4.4%, and 4.4%). High rates of moderately severe depression risk equivalence to that found among younger healthcare providers (less than 30 years) were also discovered among healthcare providers aged 30–40 (5%), and 41–50 (45%). No statistical significance between the age and severity of depression risk was found.

Findings were not significant based on years of experience (p = 0.088). The mild risk of depression was the highest (25.4%) among healthcare providers with 11–20 years of experience. Moderately severe risk of depression was also the highest (5.5%) among the same group. Severe depression risk (5.1%) was mostly noted among healthcare providers with less than 5 years of experience.

For occupation, physicians scored the highest in mild and moderate depression risks at 29.1%, and 9.1% respectively. Moderately severe risk of depression and severe risk of depression was mostly reported among nursing at 4.8% and 3%, respectively.

Among different specialties, psychiatry scored the highest in both moderately severe (10%) and severe (5%) depression risk categories. For moderate risk of depression, pediatricians exhibited the highest rate (13.7%) compared to other specialties. Although respondents from internal medicine and obstetrics and gynecology reported a higher prevalence of mild risk of depression (26.5%), however, the association between the severity of depression and specialty was not significant. As well as shown in Table 3, among various practices healthcare providers working in inpatient-based care scored highest in mild-risk depression (23.3%), however, healthcare providers working in emergency and ICU care scored higher in moderate, moderately severe, and severe depression risk categories (10.1%, 7.7%, and 4.8%, respectively), in comparing with the health care provider working in an inpatient or primary health care. but this does not reach significance with a P-value of 0.05.

**Table 3:** Depression risk severity scores and prevalence among healthcare providers in correlation with HCP gender, years of experience, age group, occupation, and specialty.

Variable	Mild depression risk % (N)	Moderate depression risk % (N)	Moderate severe depression risk% (N)	Severe depression risk % (N)	P value
**Gender**					0.119
Female HCP	23.5 (217)	9.1 9(84)	3.6 (33)	2.5 (23)	
Male HCP	19.8 (63)	8.8 (28)	6.6 (21)	3.5 (11)	
**Years of experience**					0.088
less than 5	22.7 (40)	11.9 (21)	4 (7)	5.1(9)	
6–10	18.8 (58)	10.7 (33)	3.9 (12)	2.3 (7)	
11–20	25.4 (129)	8.1 (41)	5.5 (28)	3(15)	
21–30	22.4 (44)	7.1 (14)	3.1 (6)	1.5 (3)	
more than 30	16.1(90	5.4(3)	1.8(10	0 (0)	
**Age group**					0.119
less than 30	23.9 (38)	13.2(21)	4.4(7)	4.4(7)	
30–40	22.8 (119)	10.2 (53)	5 (26)	3.1 (16)	
41–50	22.7(80)	6.5 (23)	4.5 (16)	2.3 (8)	
more than 50	20.4 (43)	7.1 (15)	2.4 (5)	1.4 (3)	
**Occupation**					0.304
Physicians	29.1(67)	9.1(21)	3 (7)	2.6 (5)	
Nursing	22.1 (171)	8.8 (68)	4.8 (37)	3 (23)	
Allied health professional	17.4 (42)	9.5 (23)	4.1(10)	2.1(5)	
**Specialty**					0.351
Family medicine	23.1 (18)	9 (7)	2.6 (2)	1.3 (1)	
Internal medicine	26.5 (26)	13.3 (13)	6.1 (6)	1 (1)	
Obstetrics and Gynecology	26.5 (27)	12.7 (13)	1(1)	2.9 (3)	
Pediatric	21.7 (30)	13.8 (19)	3.6 (5)	2.9 (4)	
Psychiatry	20 (4)	5(1)	10(2)	5(1)	
Surgery	27.7 (33)	5 (6)	4.2 (5)	3.4(4)	
Others	20.6 (142)	7.7(53)	4.8(33)	2.9 (20)	
**Practice sitting**					0.05
Emergency and ICU care	20.8 (43)	10.1 (21)	7.7(16)	4.8 (10)	
In-patient hospital	23.3 (168)	9.6 (69)	4.2 (30)	2.2 (16)	
Other	35.7 (5)	14.3 (2)	0(0)	7.1(1)	
Primary health care	21.3 (64)	6.6(20)	2.7(8)	2.3(7)	

The depression scoring system included 9 items. Each item has four responses. Each response had a certain score as the following: not at all = 0, several days = 1, more than half a day = 2, almost every day = 3. Depression items total score ranged from 0–27. A total score of 0–4 indicates minimal depression. A total score of 5–9 indicates mild depression. A total score of 10–14 indicates moderate depression. A total score of 15–19 indicates moderately severe depression. A total score of 20–27 indicates severe depression.

Linear regression for anxiety risk score showed it negatively associated with age and working in primary health care P-value of (0.002), (0.005) respectively and B = (-0.89),(-0.08) respectively. For other sociodemographic variables, no significant associations were found (Table 4A).

**Table 4:** A: Multivariate Linear Regression Analysis for Factors Associated with Anxiety symptoms (GAD7). B: Multivariate Linear Regression Analysis for Factors Associated with Depression Symptoms (PHQ9).

**Independent variable**	Beta	t	Sig.
Age	-0.89	-3.148	**0.002**
Gender	0.037	1.315	0.189
Year of experience	0.038	0.770	0.441
Emergency /ICU	.054	1.923	0.055
Primary health care	-0.08	-2.826	**0.005**
In-patient health care	0.020	1.053	0.292
Pediatric	0.033	1.181	0.238
psychiatry	0.015	0.547	0.585
Surgery	-.009	-.009	0.754
Family Medicine	0.025	-.880	0.379
Internal Medicine	0.008	0.443	0.658
Physicians	0.030	1.014	0.311
Nursing	0.006	0.291	0.771
Allied Health professional	-0.18	-0.963	0.336

**Independent variable**	Beta	t	Sig.
Age	-0.080	-4.433	**0.000**
Gender	0.037	1.315	0.189
Year of experience	0.038	0.770	0.441
Emergency /ICU	.044	1.526	0.127
Primary health care	-0.834	-2.079	**0.038**
In-patient health care	-0.018	-0.567	0.571
Pediatric	0.029	1.016	0.310
Psychiatry	0.013	0.446	0.656
Surgery	-.013	-.460	0.646
Family Medicine	0.001	-.024	0.981
Internal Medicine	0.012	0.406	0.685
Physicians	0.045	1.557	0.120
Nursing	0.004	0.122	0.903
Allied Health professional	-0.38	-1.351	0.177

Linear regression for depression risk score showed it negatively associated with age and working in primary health care, P-value of (0.000), (0.038) respectively and B = (-0.80),(-0.834) respectively. For other sociodemographic variables, no significant associations were found (Table 4B).

Comparing the anxiety mean score among different resident doctors’ specialties showed that obstetrics and gynecology residents exhibited the highest mean score for anxiety was 20 (SD 0) followed by internal medicine at 9.8 (SD 5.8). While family medicine and pediatric had lower mean scores of 3.7(SD 4) and 3 (SD 4.2).

Similar findings for mean depression scores were found to be highest among obstetrics and gynecology residents was 21 (SD 0), followed by internal medicine was 8.1(SD 2.9). While family medicine, surgery, and pediatric were 3.4 (SD 4.3), 4.5 (SD 2.1), and 3.5 (4.9) respectively.

## Discussion

The prevalence of depression and anxiety among healthcare professionals in SEHA is high during the time of the COVID-19 pandemic (38.3%, 51.5%) respectively. Results of this study are supported by systemic review and metanalysis, which relieved a pooled prevalence of anxiety is 23.2%, and a depression prevalence rate of 22.8% although this study revealed a higher anxiety prevalence [16]. A similar presence of severe anxiety was detected in another study conducted in UAE during the pandemic, however milder and moderated cases were more in this study [8]. This can be attributed to the different scales used. Que J, et al.,2020 demonstrated that the prevalence of anxiety and other psychological problems was higher among frontline healthcare workers, compared with healthcare workers who did not participate in frontline work [24]. This was further demonstrated in the present study, with severe anxiety noted among healthcare providers within the internal medicine specialty. Additionally, healthcare workers in inpatient settings revealed more depression among medical professionals than in other settings.

The incidence of moderate and severe depression was higher in males than females, Our results do not support the finding that female healthcare reported higher rates of anxiety and depression compared to males [16].

Among respondents, resident doctors experienced the highest rates of severe anxiety and severe depression, while obstetricians and gynecologists achieved the highest mean score for depression and anxiety. A study conducted in Brazil showed that psychological distress in residency training is high and related to individual and environmental factors. High workloads, the occurrence of psychological abuse, poor learning experience, poor faculty supervision, and work-personal life conflicts contribute to resident depression and anxiety [25]. Another Study showed that the prevalence of depression and anxiety was higher in the residents of the surgical branches than in those of the nonsurgical branch. This could be because of the increased workload and routine and emergency procedures [26]. In the Abu Dhabi healthcare setting, this need to be studied in a qualitative design as there was strict guidance from the DOH on residents’ participation in the COVID-19 missions and all their missions were having oversight from senior management. There may be a role to less experience and knowledge that contributed to their higher reported rates of depression and anxiety.

For resident doctors, psychological support and training are necessary to ensure their well-being, and intervention is required for obstetrics and gynecology residents.

This study only assesses depression and anxiety in the health care providers although mentioned in the systemic review that sleep problems and distress are more prevalent than depression and anxiety. Addressing sleep problems and distress in the health care provider is to be included in a future study [27].

Initiative-taking measures and mental health support are needed to ensure psychological well-being among healthcare providers [28]. Although healthcare providers in Abu Dhabi were supported by numerous measures, including the provision of psychological counseling, mental health support helplines, and psychological motivational online webinars frontline specialties should receive special attention regarding this matter. Coping with stress and establishing resilience strategies is imperative for healthcare providers. Future interventions focused on rest and social support for healthcare workers can be implemented as explained in the systemic review [27]. Abu Dhabi was rated among the best-performing cities in the world regarding the preparedness and supportive measures among them, including the healthcare system [29]. Nevertheless, the mental health of the healthcare workers seems an area worth investing in with the high depression and anxiety found. In interventional studies conducted in the United Kingdom (UK), the provision of an e-package for healthcare workers that includes evidence-based guidance, support, and signposting related to psychological well-being was mandated for healthcare employees [30]. This study can be the first step in preparing an action plan to support the healthcare workers’ mental health, but designing any intervention will need a qualitative study to ensure a successful outcome. A vital component of the disaster management of infectious pandemics is to address the psychological effects on healthcare providers [31].

## Conclusion

HCP experienced a high prevalence of anxiety and depression during the COVID-19 pandemic, suggesting that mental health intervention and support are necessary for HCP to ensure the psychological well-being of healthcare providers.

### Limitations of the study

The participants in this study primarily originated from Abu Dhabi city and represent the Abu Dhabi healthcare population. This, therefore, limited the generalizability of the study findings, although the respondents originated from both, the public and private sectors. Social desirability bias may have occurred because the questionnaire was self-administered. However, the anonymity of the questionnaires was maintained. Despite limitations, the study provides a reasonable source of information and was developed and conducted within a very short time frame this can provide a timely response for identified risks and challenges and also the chance to assess any implemented intervention. Follow-up studies to assess the progression of the psychological impact of the COVID-19 pandemic are needed. No medically recorded review was accomplished for the health care providers, and social factors and support were not measured in this study. The timing of the study is at the earliest stages of the pandemic, which may have differed later.

## Abbreviations

COVID-19: Coronavirus Disease-2019
WHO: World Health Organization; and Practices
HCW: Healthcare Worker
HCPs: Healthcare providers
HCP: Healthcare Professionals
PHQ-9: Patient Health Questionnaire-9
GAD 7: Generalized Anxiety Disorder -7
